# A Fiber Bragg Grating-Based Anemometer †

**DOI:** 10.3390/s18072213

**Published:** 2018-07-10

**Authors:** Chuan-Ying Huang, Pei-Wen Chan, Hung-Ying Chang, Wen-Fung Liu

**Affiliations:** 1 Program of Electrical and Communications Engineering, Feng-Chia University, Taichung 40724, Taiwan; hungying.chang@gmail.com; 2 Department of Electrical Engineering, Feng-Chia University, 100 Wen-hwa Rd., Seat-wen, Taichung 40724, Taiwan; bunny523119@gmail.com (P.-W.C.); wfliu@fcu.edu.tw (W.-F.L.)

**Keywords:** anemometer, stainless steel pipe, wind-speed measurement, wind-direction measurement, fiber Bragg grating (FBG)

## Abstract

A novel fiber anemometer based on two pairs of fiber gratings is experimentally demonstrated and can simultaneously detect wind speed and wind direction. One pair of gratings, which are separated by 90° in space, is fixed on a small stainless steel pipe driven by a rotating disc for measuring the wind-direction angle. The other pair is composed of a sensing and a matched grating. The frequency of the spectrum-shifted of the sensing grating to overlap with that of the matched grating is employed for determining the wind speed. The errors in the wind-speed and wind-angle measurements are experimentally demonstrated to be less than 1%. The proposed fiber anemometer with a simple and durable structure can be applied in wind-powered electricity generators.

## 1. Introduction

Due to global warming and climate change, the renewable energy contribution of wind power is growing in demand in all countries. The most crucial parameter in wind-powered electricity generators is the wind speed magnitude, which for safety should be monitored in real time by using an anemometer. Many distinct types of traditional anemometers—such as cup, vane, hot wire, laser Doppler, ultrasonic, table tennis, pressure, plate, and pitot-static anemometers [1]—are commercially available and have been used in practical wind-speed measurements. These sensors have a simple and reliable structure, strong wind resistance, linear response, fast response, and high accuracy. However, they are susceptible to electromagnetic interference. Additionally, the power supply stability of wind gauges is very important for long-term operation because under unstable conditions, an anemometer may work discontinuously and deliver incorrect wind information. To solve these problems, anemometers based on optical fiber techniques have been proposed [2,3,4,5,6], among which the fiber grating anemometer is an essential sensing technology due to its wide range of applications [7,8,9,10] and optical characteristics, such as immunity to electromagnetic interference, high sensitivity, small size, lightness, long lifetime, and corrosion resistance. The fiber Bragg grating (FBG) [11] is a passive optical component with periodic index modulation along the fiber axis. Due to its high repeatability and stability, the phase mask writing technique is commonly used for fabricating fiber gratings. It employs a 248 nm KrF excimer laser as the writing light source. The excimer laser beam passes through the phase mask to create diffracted light, and a commercial communication fiber (SMF-28) is exposed to the interference pattern from ±1st order diffraction light to form an FBG. An amplified spontaneous emission (ASE) broadband light source, a 3-dB coupler, and an optical spectrum analyzer are used for monitoring fiber grating growth in real time. The grating wavelength (λ_B_) equals to 2*n_eff_Λ*, where *n_eff_* is the grating effective index and *Λ* is the grating period. Thus, the center wavelength of a fabricated grating can be determined by the period of the phase mask because this period is double that of the grating.

Previously proposed fiber grating anemometers [12,13] only measure wind speed and cannot simultaneously measure the wind-direction angle. Thus, by extending the temperature-independent rotation angle of the fiber grating sensor [14], we propose an anemoscope comprising two pairs of fiber gratings. This anemoscope simultaneously measures the wind speed and wind-direction angle. In order to measure the wind-direction angle, the two fiber gratings separated by 90° glued on a small stainless steel pipe are compressed or stretched by the wind force to cause the grating wavelength shift. In order to measure the wind speed, the frequency of the wavelength shift of the sensing grating to be overlapped with that of the matching fiber grating is obtained. The proposed fiber grating anemometer can be applied in wind power generators, the defense industry, civil aviation, transportation, factory production, and meteorology.

## 2. Sensing Principle and Sensing Heads

**Figure 1:**
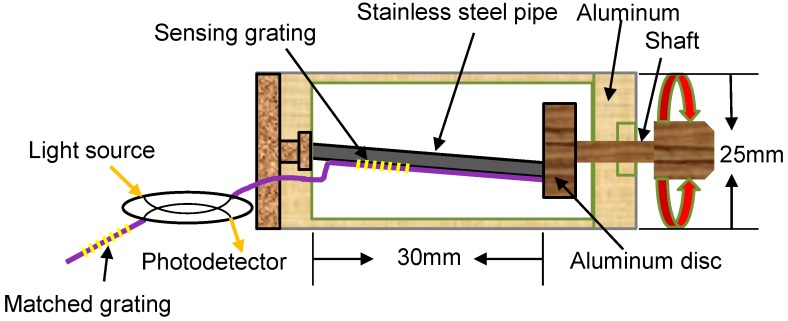
Configuration of the wind-speed sensing head.

Because an FBG is the key component of the proposed fiber anemometer, the sensing mechanism is based on the strain induced by the wind force, which causes a grating wavelength shift. The function of the FBG is similar to that of a narrow-bandwidth fiber mirror with a central reflection wavelength (*λ_B_* = 2*n_eff_ Λ*). When a broadband light source is launched into one end of an FBG, a narrow-bandwidth reflection spectrum is produced. The grating transmission spectrum can be observed at the other end of the FBG. If an axial strain or temperature variation is applied to the grating, the grating wavelength is shifted. Thus, the relationship between the grating wavelength shift (*Δλ_B_*) and axial strain (*ε*) applied to the FBG is given as follows [15]:

(1) $$\Delta \lambda_{B}/\lambda_{B}=\left(1-P_{e}\right)\varepsilon$$

where *P_e_* indicates the effective photo-elastic coefficient of the glass fiber. For a typical fused-silica fiber, *P_e_* is approximately 0.22 under longitudinal stress. Thus, in accordance with Equation (1), we design the sensing head for measuring the wind speed by using a pair of fiber gratings including a sensing grating and a matching grating as shown in Figure 1.

The sensing grating glued on the steel pipe head is shielded by an aluminum cylinder with a diameter of 25 mm. The aluminum shaft with a disc is driven by rotating a fan and is used for detecting wind speed. One end of the 1.5 mm-diameter steel pipe is fixed axially at the center of the nonshaft side of the aluminum cylinder, and another lead is mounted on the aluminum disc. A 20 mm-long shaft is extended inside the cylinder for being bonded with a 7 mm-diameter aluminum disc.

**Figure 2:**
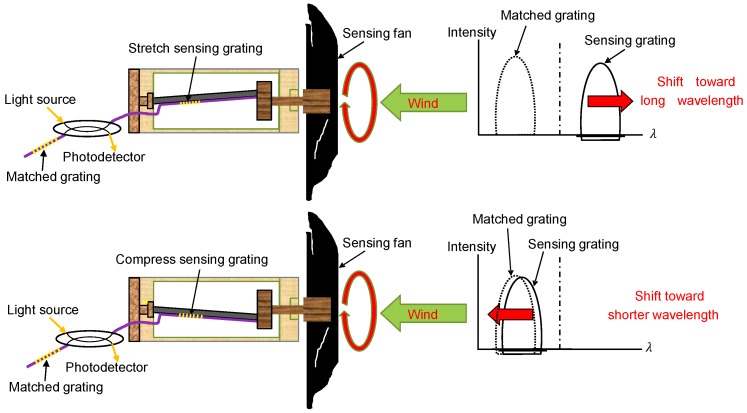
Sensing concept for measuring wind speed.

**Figure 3:**
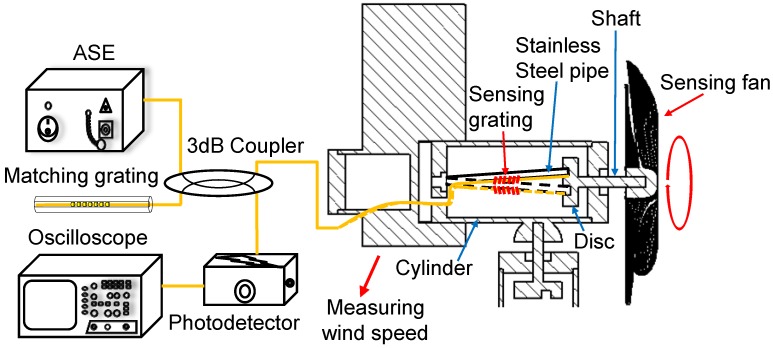
Experimental setup of the sensing head for measuring wind speed.

The sensing mechanism of the sensing head is illustrated in Figure 2. [16] One edge of the disc is fixed to the end of the small stainless steel pipe, and the other edge is attached to the rotating shaft, which is extended axially through the cylinder. The other end of the rotating shaft is connected to a rotational fan, and the rotating shaft with the disc is synchronously rotated by the fan. When the fan is rotated, the sensing grating fixed on the steel pipe surface is stretched and compressed, which causes the grating wavelength to shift back and forth. Thus, if the original central wavelength of the matching grating is appropriately selected, the spectrum of the sensing grating shifts back and forth with the rotation of the fan, which creates a spectrum-overlapped frequency to be detected by using a photodetector as shown in Figure 3. An oscilloscope is employed to monitor the spectrum-overlapped frequency, which can then be used to determine the wind speed.

**Figure 4:**
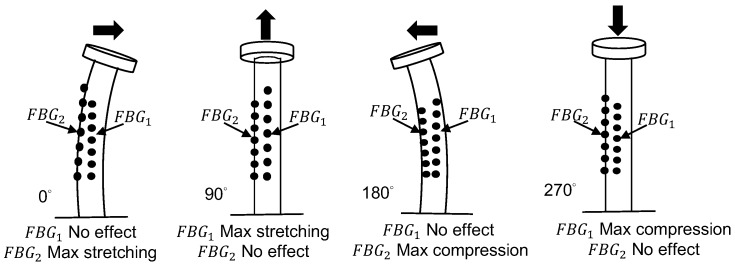
Sensing concept for measuring wind direction.

The sensing concept used for measuring wind direction is illustrated in Figure 4. Because the two FBGs with different central wavelengths are bonded to the small stainless steel pipe surface at a 90° separation, the shifts of the maximum and minimum central wavelengths are obtained when the steel pipe is rotated in the range from 0° to 360°. Therefore, the wind angle is identical to the rotation angle. By using the arc tangent function, we can determine the wind direction from the following three equations:

(2) $$\theta_{tan}=tan^{-1}(\frac{\Delta \lambda_{1}}{\lambda_{01}}/\frac{\Delta \lambda_{2}}{\lambda_{02}})$$

(3) $$\Delta \lambda_{1}=\lambda_{FBG1}-\lambda_{01}$$

(4) $$\Delta \lambda_{2}=\lambda_{FBG2}-\lambda_{02}$$

where $\lambda_{01}$ and $\lambda_{02}$ are the original central wavelengths of *FBG1* and *FBG2*, respectively; and *λ_FBG1_* and *λ_FBG2_* represent the shifted central wavelengths of *FBG1* and *FBG2*, respectively, when the small stainless steel pipe is rotated. Due to $\theta_{tan}$ in Equation (2), the angle can only have the range from −90° (270°) to 90°. To obtain an overall expression from 0° to 360°, Equation (2) can be modified as follows:

(5) $$\theta =\theta_{tan}+\left[\left(1-\left|\frac{\Delta \lambda_{2}}{\lambda_{02}}\right|/\frac{\Delta \lambda_{2}}{\lambda_{02}}\right)\times \frac{\pi }{2}\right]+\frac{\pi }{2}$$

**Figure 5:**
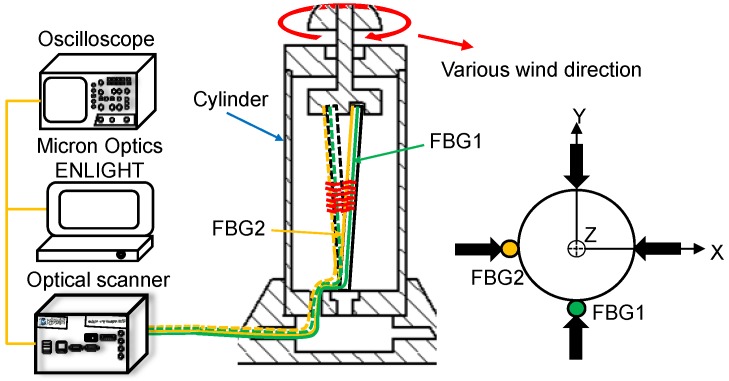
Experimental setup for measuring wind direction.

The experimental setup for measuring the wind direction is depicted in Figure 5, where the two FBGs glued on the stainless steel pipe are the crucial components for detecting the wavelength shift. The two FBGs are separated by 90°. One end of the stainless steel pipe is fixed on the base, and the other is connected to a rotating disc. Under different wind directions, the disc bends the stainless steel pipe by different angles. The two FBGs are stretched or compressed with different magnitudes, which results in different wavelength shifts. Thus, the wind direction can be determined from the wavelength shifts of the two gratings.

## 3. Experimental Setup and Results

**Figure 6:**
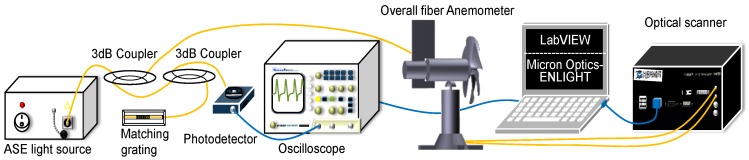
Experimental setup for measuring both wind speed and direction.

The overall experimental setup, including the fiber anemometer for measuring wind speed and direction, is displayed in Figure 6. For the sensing head used in wind-speed measurement, the central wavelength of the matching FBG is equal to the maximum wavelength shift of the sensing grating. Because the sensing grating is stretched by the stainless steel pipe, the grating wavelength is shifted toward the matching FBG, which creates an overlapped optical signal. The overlapped signal is reflected to the power meter, and the overlapped frequency is detected using an oscilloscope. By contrast, when the central wavelength of the sensing grating is outside the overlapped region, the power meter cannot detect any reflective signal. For measuring wind direction, while the shaft is being rotated by the rotor, the central wavelengths of the two FBGs are shifted and display sinusoidal variation with a phase difference of 90° between them. The rotation angle of the rotor is equivalent to the wind-direction angle. Thus, an optical scanner [17] is used, with its internal light source connected to the fiber anemometer, to monitor the wavelength shift in the FBGs for obtaining the corresponding shaft angle variation and detecting wind direction.

**Figure 7:**
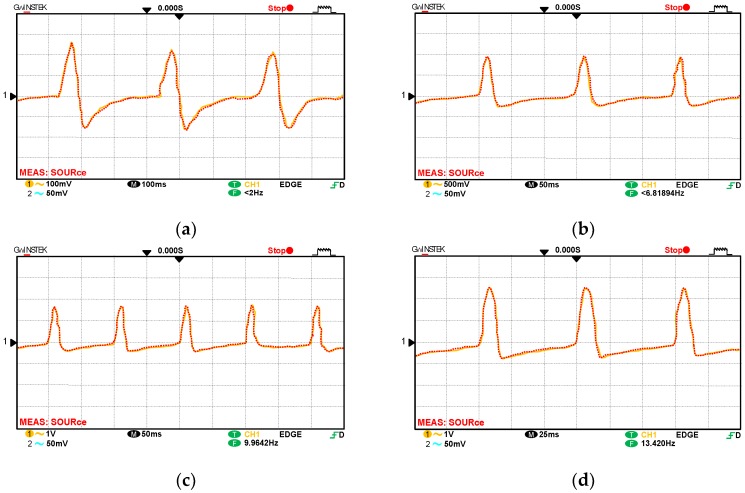
Oscilloscope diagrams for wind speeds (**a**) 200; (**b**) 400; (**c**) 600; and (**d**) 800 rpm.

**Figure 8:**
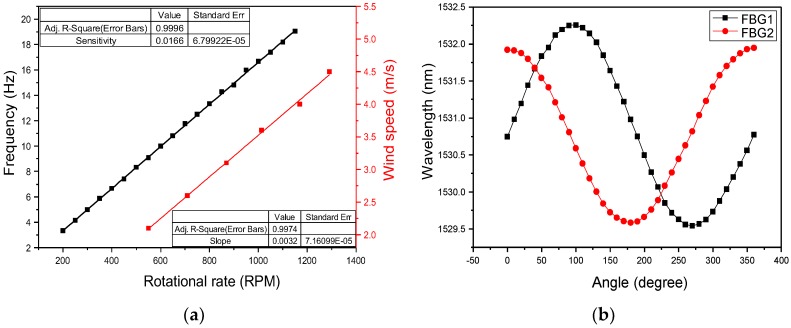
Plots of (**a**) frequency and wind speed versus rotational speed and (**b**) grating wavelength shift versus wind-direction angle.

For the wind speed experiment, the frequency diagrams obtained in the oscilloscope with four rates (200, 400, 600, and 800 rpm) are displayed in Figure 7. Figure 7 indicates that the accuracy of the overlapped frequency can be obtained. The black line in Figure 8a indicates the overlapped frequency when the rotational speed rate is increased from 200 to 1150 rpm in increments of 50 rpm. This performance can be confirmed using a commercial electric fan with various wind-speed settings for blowing the fiber anemometer. The red line in Figure 8a represents the relationship between wind speed and the speed rate of the sensing fan. To confirm the accuracy of the proposed fiber anemometer, the experimental results are compared with the measured values by using a standard wind-speed meter, an uncertainty in wind speed of less than 1% is obtained.

The plots of frequency and wind speed versus the rotational speed rate of the sensing fan are displayed in Figure 8a, where the slope of the black line is 0.0166. The relationship between the frequency (Hz) and rotational speed rate (*R*) can thus be described using the following equation:

(6) R=60.24f

where R is the rotational speed rate in RPM of the fan and f is the rotational frequency in Hz. By using Equation (6), the rotational speed can be calculated from the frequency measured by the oscilloscope. The slope of the red curve in Figure 8a is 0.0032. Thus the relationship between the wind speed and rotational speed rate can be described using the following equation:

(7) S=0.0032R+0.323

where S is the tested wind speed in m/s, R is the rotational speed rate of the sensing fan, and 0.323 is the compensation coefficient obtained from the experimental data. By using Equation (7), the tested wind speed can be calculated from the rotational speed rate. By substituting Equation (6) into Equation (7), the relationship between the measured frequency and wind speed can be described by the following equation:

(8) S=0.1928f+0.323

For measuring the wind direction, two FBGs separated by 90° and having different central wavelengths are bound axially to a stainless steel pipe to form a wind-direction sensing head. When the wind direction is changed from 0° to 360°, in increments of 10°, the locations of the maximum and minimum central wavelength of the gratings are separated accordingly. From the experimental data, the curves of the wavelength shifts of the two FBGs for various wind-direction angles are displayed in Figure 8b. From Figure 8b, the wind-direction angle can be obtained from the following equation:

(9) $$\theta =\left(\cos^{-1}\frac{FBG2-\lambda_{02}}{\lambda_{max2}-\lambda_{02}}\right)\times \frac{FBG1-\lambda_{01}}{\left|FBG1-\lambda_{01}\right|}+\left(1-\frac{FBG1-\lambda_{01}}{\left|FBG1-\lambda_{01}\right|}\right)\times 180˚$$

where θ is the wind direction, FBG1 and FBG2 are the two measured grating wavelengths, $\lambda_{01}$ and $\lambda_{02}$ represent the original central wavelengths, and $\lambda_{max2}$ is the maximum wavelength shift for the *FBG2* grating. Based on the aforementioned equation, LabVIEW and Micron Optics-ENLIGHT™ are used to determine the rotational frequency and wavelength shift. Thus, the testing wind speed and wind direction angle can be simultaneously obtained.

**Figure 9:**
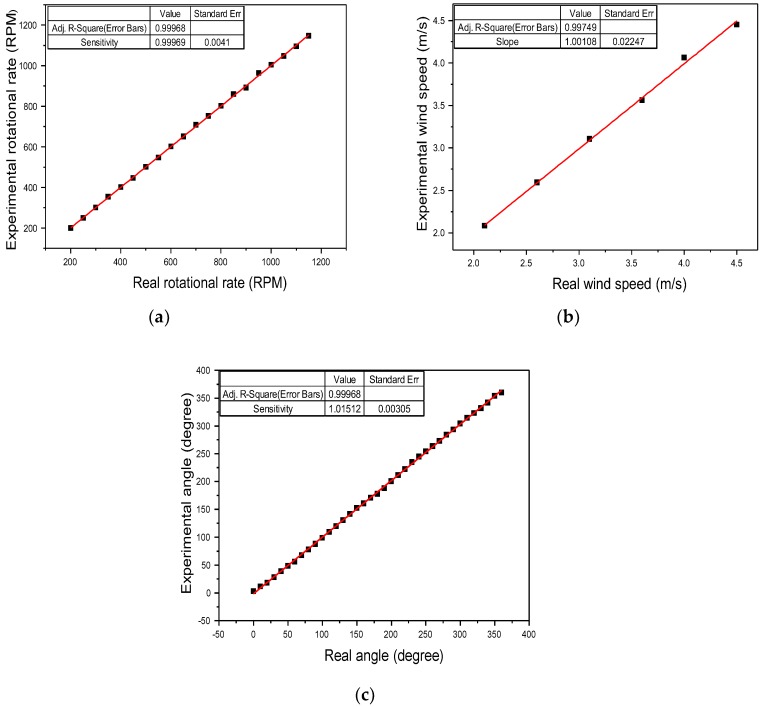
Relationship curves between the real and experimental (**a**) rotational speed, (**b**) wind speed, and (**c**) wind-direction angle.

A comparison between the experimental results and real values is illustrated in Figure 9. For the proposed fiber anemometer, the measurement errors for wind speed and wind-direction angle are less than 1%.

## 4. Conclusions

In this paper, a fiber anemometer with two pairs of FBGs is designed that can simultaneously measure wind speed and direction. One FBG pair comprises a sensing grating and matching grating for wind-speed measurement. The other pair comprises two gratings with different central wavelengths for wind-direction measurement. Due to its favorable performance and advantages such as small size, long-term stability, and immunity to electromagnetic interference, the proposed fiber anemometer can be used in a wide range of applications in various environments.
